# Miniaturized Bio-and Chemical-Sensors for Point-of-Care Monitoring of Chronic Kidney Diseases

**DOI:** 10.3390/s18040942

**Published:** 2018-03-22

**Authors:** Antonio Tricoli, Giovanni Neri

**Affiliations:** 1 Nanotechnolgy Research Laboratory, Research School of Engineering, Australian National University, Canberra ACT 0200, Australia; antonio.tricoli@anu.edu.au; 2 Department of Engineering, University of Messina, I-9866 Messina, Italy

**Keywords:** POC testing devices, kidney disease, chemical sensors, biosensors

## Abstract

This review reports the latest achievements in point-of-care (POC) sensor technologies for the monitoring of ammonia, creatinine and urea in patients suffering of chronic kidney diseases (CKDs). Abnormal levels of these nitrogen biomarkers are found in the physiological fluids, such as blood, urine and sweat, of CKD patients. Delocalized at-home monitoring of CKD biomarkers via integration of miniaturized, portable, and low cost chemical- and bio-sensors in POC devices, is an emerging approach to improve patients’ health monitoring and life quality. The successful monitoring of CKD biomarkers, performed on the different body fluids by means of sensors having strict requirements in term of size, cost, large-scale production capacity, response time and simple operation procedures for use in POC devices, is reported and discussed.

## 1. Introduction

Current medical diagnostics work-up and monitoring of many diseases by conventional diagnostic analysis in laboratory and hospital is perceived as invasive, time consuming and expensive. Point-of-care (POC) technology for testing and monitoring of biomedical markers is a promising approach to: (i) decrease cost, save time and reduce complexity of analysis; (ii) enable close-to-home patient testing in medical centers and (iii) enable deliver of home healthcare services. However, even when highly precise, many current clinical methods/techniques for biomarkers analyses have some limitations in terms of cost, size and integration into portable point-of-care medical devices. In this context, electrochemical detection systems can offer high sensitivity, low-cost, strong miniaturization potential and easy integration in compact analytical devices [1]. Emerging POC devices with miniaturized sensors based on different electrochemical techniques (e.g., potentiometric, amperometric) have the potential to supply real time information for the diagnosis and management of several diseases via self-analysis of physiological fluids such as blood, urine and sweat [2,3]. Electrochemical biosensors are inexpensive, mass-produced disposable devices. Miniaturisation and use of only one drop of a few microliters of body sample is another factor that enable their use to be moved to the POC setting. 

Depending on the nature of the sensing element, electrochemical sensors are usually categorized in enzymatic sensors and enzyme-free sensors [4]. The former sensors use an enzyme (e.g., urease, creatininase) to assist the sensing mechanism. The most important advantage is the almost exclusive selectivity to the target analyte [5]. 

Recent R&D into the optimization of enzyme-free sensors is showing some promising results. The latter sensors are based on the use of inorganic and/or organic sensing materials. Over the last decade, molecularly imprinted polymers (MIPs) have attracted much attention as highly promising artificial biomolecular recognition materials thanks to their versatility and capability to incorporate binding sites for various types of analytes [6]. Sensors based on MIPs present high selectivity, inherent stability under relatively harsh conditions, long shelf life and low cost. However, MIPs-modified sensors typically suffer from low sensitivity, due to their poor conductivity and electro-catalytic activity. Electroactive metal and metal oxides have also been increasingly investigated [7], due to their very high sensitivity and stability. In addition, these devices have reduced chance of causing exposure to biohazards. However, their selectivity is still often insufficient. In summary, the design of enzyme-free devices is promising in clinical use, as well as diagnostic monitoring for POC applications.

Other electrical transduction-based sensor technologies are demonstrating the potential to measure reliably numerous biomarkers in decentralized settings. For instance, in the last decade, the biomedical applications of conductometric gas sensors for breath analysis have been greatly improved [8]. 

All these emerging technologies offer several benefits over conventional diagnostic analysis of biomarkers in breath, blood and urine, including simplicity of use, specificity for the target analyte, short diagnostic/measurement time, capability for continuous monitoring and multiplexing, together with the potentiality of integration with low-cost, portable instrumentation. So, they have been proposed in the last decade for monitoring clinical biomarkers for many diseases.

Amongst chronic diseases, kidney malfunctioning is one of the most severe and involves a large fraction of the world population [9]. Patients with end stage renal disease, i.e., suffering of chronic kidney disease (CKD), have limited mobility, and must go to the hospital for diagnosis and care. 

**Figure 1:**
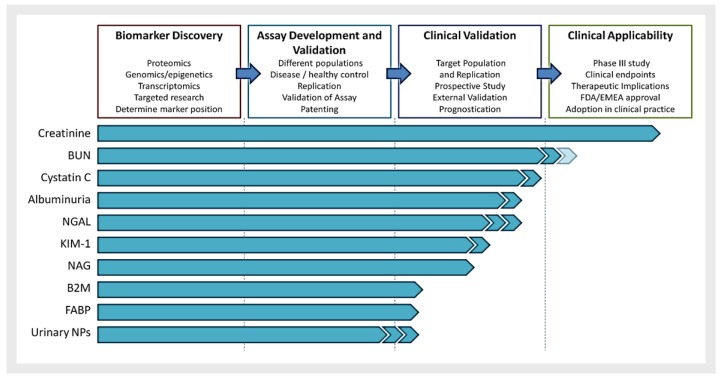
Renal biomarker development. Approach to renal biomarker discovery and clinical applicability. B2M, b-2-microglobulin; BUN, blood urea nitrogen; FABP, fatty acid-binding protein (types L and H); KIM-1, kidney injury molecule 1; NAG, *N*-acetyl-β-D-glucosaminidase; NGAL, neutrophil gelatinase-associated lipocalin; NP, natriuretic peptide. Reprinted from [10].

The determination of precise biomarkers for this pathology has, however, not been straightforward [10]. A series of biomarkers have been proposed (see Figure 1).

Many of these are suitable only to be monitored in specialized laboratory and/or are still under evaluation. Abnormal levels of nitrogen end products of metabolism such as creatinine and urea are presents in the blood of CKD patients, and therefore have been considered, since long, as important biomarkers for these pathologies. These biomarkers are the simplest to measure also from not specialized operators or the patient itself and are therefore promising for use in POC devices. Measurement of blood urea nitrogen (BUN) and serum creatinine (sCr) levels are the most used screening tests of renal function. BUN is about one-half (28/60 or 0.446) of blood urea level. The normal range of urea nitrogen in blood or serum is 5 to 20 mg/dL, or 1.8 to 7.1 mmol urea per liter. Patients with end-stage renal failure, requiring dialysis or renal transplantation, may have plasma/serum urea above 50.0 mmol/L (BUN > 140 mg/dL). Urea clearance is used for fixing appropriate timing of dialysis [11]. 

Recently, plasma creatinine estimation has emerged as the preferred test for assessment of renal function. Creatinine is the product of muscle creatine catabolism. The normal serum creatinine (SCr) for an adult male is in the range 0.6 to 1.2 mg/dL, or 53 to 106 μmol/L and slightly lower (~10%) for adult females, due to the lower muscle mass [12]. As creatinine is produced in the body at virtually constant rate, and its concentration in the blood changes little, creatinine clearance based on serum creatinine levels is usually used as a measure of renal function, calculated by Cockroft-Gault formula [13]. The potential use of blood ammonia as a biomarker in non-invasive POC testing, has been also recently demonstrated [14]. 

Conventional methods to measure these biomarkers require blood sampling in mL volumes. As they are also found in other physiological fluids such as breath, urine and sweat, the search for new sensors for assessing the levels of these biomarkers in a more comfortable way is focus of broad research efforts. Breath measurement is likely the most non-invasive method in this respect. Non-invasive breath testing of ammonia has been proposed to help patients under dialysis treatment to prevent well-known detrimental side effects of renal replacement therapy [15]. Urine is the other physiological fluid used since many years to detect certain metabolic disorders, such as urinary tract infections. Since urea is the most abundant organic solute in urine, it was one of the first substances to be determined for evaluating kidney diseases. The urine urea nitrogen tests how well the kidneys are functioning. Measurement of urinary urea has been also suggested as a means of estimating nitrogen balance in hospitalized patients who are malnourished [16]. At last, compared to other biofluids, sweat is one of the most available and simple candidates for routine analysis tests [17]. Sweat is a clear hypotonic fluid containing sodium, chloride, potassium, urea, lactate, bicarbonate, calcium, ammonia, organic and non-organic compounds [18]. However, the process of collection, transportation, and estimation of biomarkers in the sweat is more complicated than blood or urinary tests and thus not commonly used. Challenges include very low sample volumes (nL to µL), unknown concentration due to evaporation, filtration and dilution of large analytes, mixing of old and new sweat, and the potential for contamination from the skin surface [19]. However, notwithstanding these present limitations, it is expected that in the future sweat analysis could extensively used in POC devices [20]. 

The benefits for patients coming from these alternative body fluids for analysis, are substantial. For instance, they could allow monitoring of health status in real time to meet therapy goals. They also provide a more user-friendly alternative than blood analysis, which is of particular benefit for the diagnostics of children and other vulnerable subjects.

This review presents a summary of the latest developments and achievements in chemical- and bio-sensors for renal diseases testing, focusing on sensor design and analytical performances. The state-of-the-art on the use of ammonia, urea and creatinine levels as clinical biomarkers for renal diseases is discussed, with focus on emerging and future chemo- and biosensor devices for POC testing. Notably, the rapid improvement in miniaturized sensing technologies has significantly increased the potential of these devices to reduce the cost of medical diagnostics, while providing better quality health monitoring for CKD patients. 

## 2. Urea

### 2.1. Blood Urea 

Blood urea analysis is routinely performed in clinical laboratories. Urea present in blood originates from the breakdown of protein from the food and body metabolism. This nitrogen compound is removed from blood by the kidneys. Urea is therefore an important marker for renal function. Under renal failure level of urea in serum increases up to 100 mM (the normal range is from 1.7 to 8.3 mM). Blood urea nitrogen is also a simple clinical variable that provides useful prognostic information in patients admitted for decompensated heart failure [21]. 

Several types of electrochemical bio-sensors are used for the quantification of blood urea levels. Enzymatic biosensors are based on urease, an enzyme catalyzing the hydrolysis of urea, anchored on the working electrode [22]. Use of urease as a probe for estimation of urea in biological samples has the advantage to provide high selectivity towards urea. Ma et al. presented a portable low-power battery-driven bioelectrochemical signal acquisition system for urea measurement [23]. This is a low cost, highly portable system. The sensor was fabricated by embedding urease enzyme into the polymer (aniline-co-o-phenylenediamine) matrix and then depositing it onto a MEMS-fabricated Au working electrode. A linear correlation was observed between the urea concentration and the electrode potential within a concentration range from 3.16 × 10^−4^ to 3.16 ×10^−2^ M with a sensitivity of 31.12 mV/log [M] and a R2 precision of 0.995. This portable device was able to operate continuously for more than four days with a 3.7 V rechargeable lithium-ion battery of 500 mAh showing a significant potential for home-care applications.

Recently, a planar potentiometric urea biosensor was demonstrated for non-invasive monitoring of BU level via reverse ionophoresis [24]. This device displayed sensitivity of 35.07 mV/decade and a linear response from 10 μM to 5 mM urea. The sensor performances were unaffected by the presence of some interference ions, such as Cl^−^, K^+^, Na^+^ and Mg^+^. The sensor performances were further validated by clinical investigation of human subjects, establishing a correlation between BU level and transdermal extracted urea level.

Eggenstein et al. reported an urea-sensitive biosensor using a NH_4_^+^-sensitive disposable electrode in double matrix membrane (DMM) technology as potentiometric transducer [25]. The sensor responded rapidly and robustly to changes in urea concentrations between 7.2 × 10^−5^ and 2.1 × 10^−2^ mol/L. The detection limit was 2 × 10^−5^ mol/L urea and the slope in the linear range 52 mV/decade. By accounting for the cross-sensitivity to the interfering K^+^- and Na^+^-ions, the sensor can be used for the determination of urea in human blood and serum samples (diluted or undiluted). 

However, such enzymatic sensors have significant stability issues, due to the denaturation of the urease enzyme. Various metal oxides or metal/metal oxide composites, have been investigated for the detection of urea. Among them, Ni-based catalyst presents better performance in the electro-catalytic oxidation of urea. Arain et al. implemented NiO nanostructures for developing a highly sensitive, simple, selective, and stable urea sensor without using any urease, in biological samples such as blood, urine, and duodenal fluids, and needs no specific storage conditions [26]. Silver catalyst deposited on ZnO rods/carbon substrates were also proposed as enzyme-free urea sensors [27]. 

### 2.2. Urine Urea

**Figure 2:**
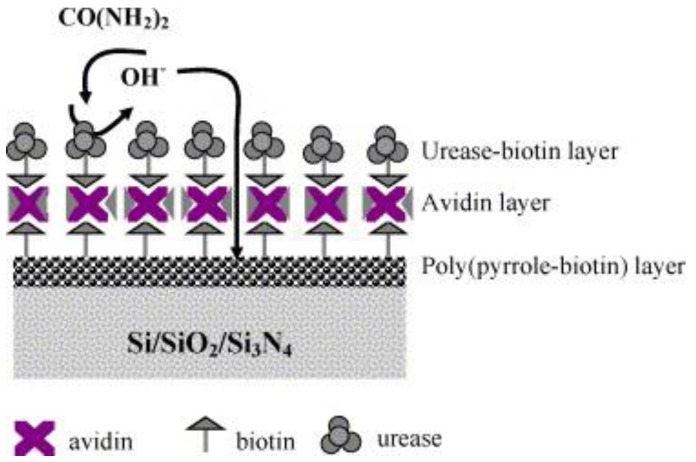
Schematic of the assembled multi-layer system. Reprinted from [28].

Urea is a waste product of the body. Healthy kidneys filter urea and remove it from the blood. The filtered waste-urea leaves the body through urine, so, as kidney function decline, urinary urea also decline. The determination of urinary urea is widely used as screening test for the evaluation of kidney function, status, and response to treatment. In recent years, extensive efforts have been dedicated to find an optimized strategy for urease immobilization in the aim to develop commercial urea enzymatic biosensors. Maaref et al. presented a study on support materials for urease immobilization based on organic and inorganic matrices [28]. With aim to obtain good reproducibility for further urea biosensors development, the matrices containing urease biomolecules were deposited on insulator semiconductor (IS) structures by a spin-coating process (Figure 2). The enzyme electrode exhibits good performances, exhibiting a linear response in the range from 10^−4^ to 10^−1^ M. 

**Figure 3:**
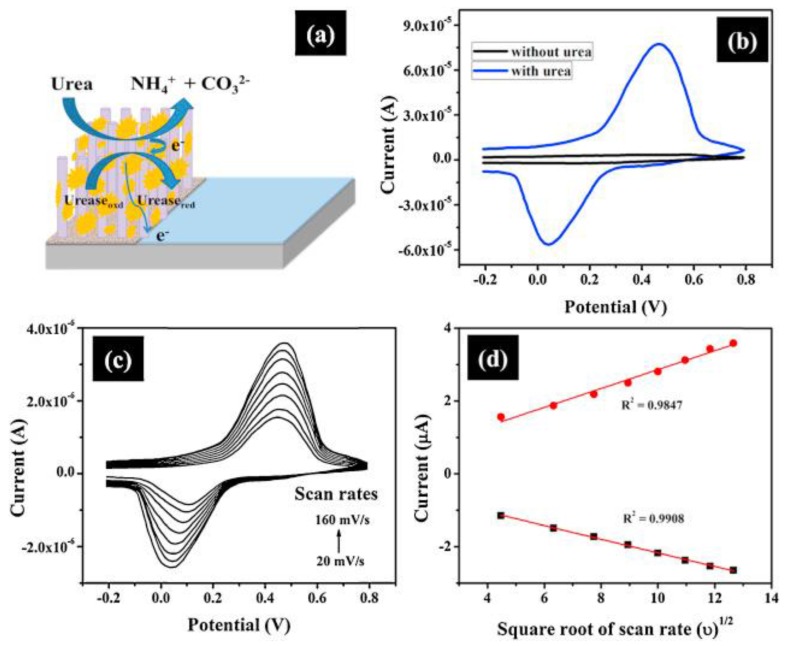
(**a**) Schematic drawing of a prototype sensor (glass/Ag/ZnO NRs/urease) for urea sensing, (**b**) CVs of urea sensor in absence and presence of 0.5 mM urea at 50 mV/s scan rate in PBS (pH 7); (**c**) CVs at different scan rates, 20–160 mV/s, and (**d**) magnitudes of peak oxidation (*I*_pa_) and reduction (*I*_pc_) currents as a function of (scan rate)^1/2^. Reprinted from [29].

Urease catalyzes hydrolysis of urea to carbamine acid that further gets hydrolyzed to ammonia (NH_3_) and carbon dioxide (CO_2_). The electrons generated from these biochemical reactions are transferred to the electrode, which amplifies the electrochemical signal resulting in improved sensor sensitivity [29]. The electrochemical process and the electrical signal of the sensor generated in different experimental conditions are shown in Figure 3. 

Non-enzymatic sensors for urea determination in urine have been also proposed. A NiCo_2_O_4_/3D graphene electro-catalyst was synthesized for the non-enzymatic detection of urea in urine samples. The NiCo_2_O_4_/3D graphene/ITO sensor exhibited a sensitivity of 166 μAm M^−1^ cm^−2^, a linear range of 0.06–0.30 mM and a fast response time of approximately 1 s with a detection limit of 5.0 µM [30]. Ansari described an enzyme-less urea sensor based on nano-tin oxide synthesized by hydrothermal technique for urea sensing from 1 to 20 mM. The sensitivity is estimated as 18.9 μA/mM below 5 mM and 2.31 μA/mM above 5 mM with a limit of detection of 0.6 mM [31].

### 2.3. Sweat Urea

Urea is one of the major analytes present in the sweat, then its determination via this body fluid is currently largely investigated. Sweat urea nitrogen of CKD patients is increased when compared with healthy subjects. Urea concentrations in the sweat of patients at the final stage of renal disease were found to be consistently much higher than the serum levels, reaching in some cases 50 times the serum level [32]. 

**Figure 4:**
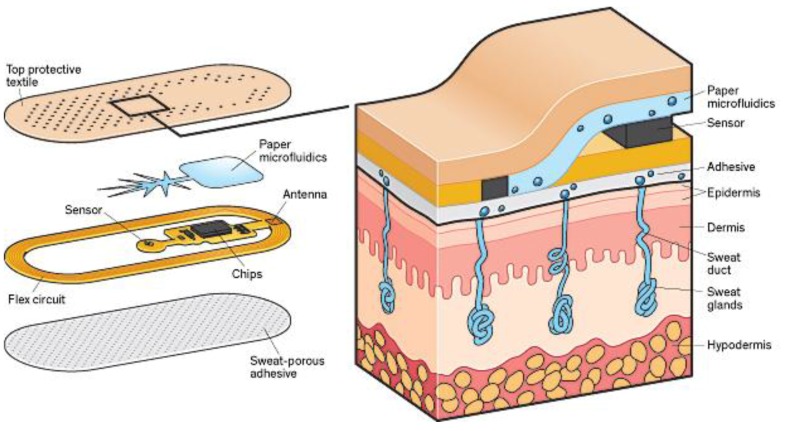
Patch developed at the University of Cincinnati using paper microfluidics to wick sweat from the skin through a selective membrane. Onboard circuitry calculates the ion concentration and sends the data to a smartphone. The electronics within the patch are externally powered, as in an RFID chip. Reprinted from [33].

Sample recording is one of the major challenges of sweat analysis. Furthermore, the procedure is time-consuming, complex, and subject to errors due to contamination. However, recently, researchers from the University of Cincinnati and the U.S. Air Force Research Laboratory, have developed patches that stimulate and analyze sweat and then wirelessly relay data to a smart-phone, which will help in accelerating the commercialization of sweat sensor technologies (Figure 4). 

Northeastern’s Center for High-rate Nanomanufacturing (CHN) has developed a simple and highly sensitive multi-biosensor containing semiconductor single-walled carbon nanotubes (SWCNTs) that are enzyme-immobilized for detecting d-glucose, l-lactate, and urea in sweat. The utilization of semiconducting carbon nanotubes for electric detection results in high repeatability and sensitivity. By leveraging the advantage of the carbon nanotubes’ electrical response and enzyme reaction, fast, specific, and continuous detection is achieved. Printing of nanomaterials to create the sensor results in low manufacturing cost [34]. 

**Figure 5:**
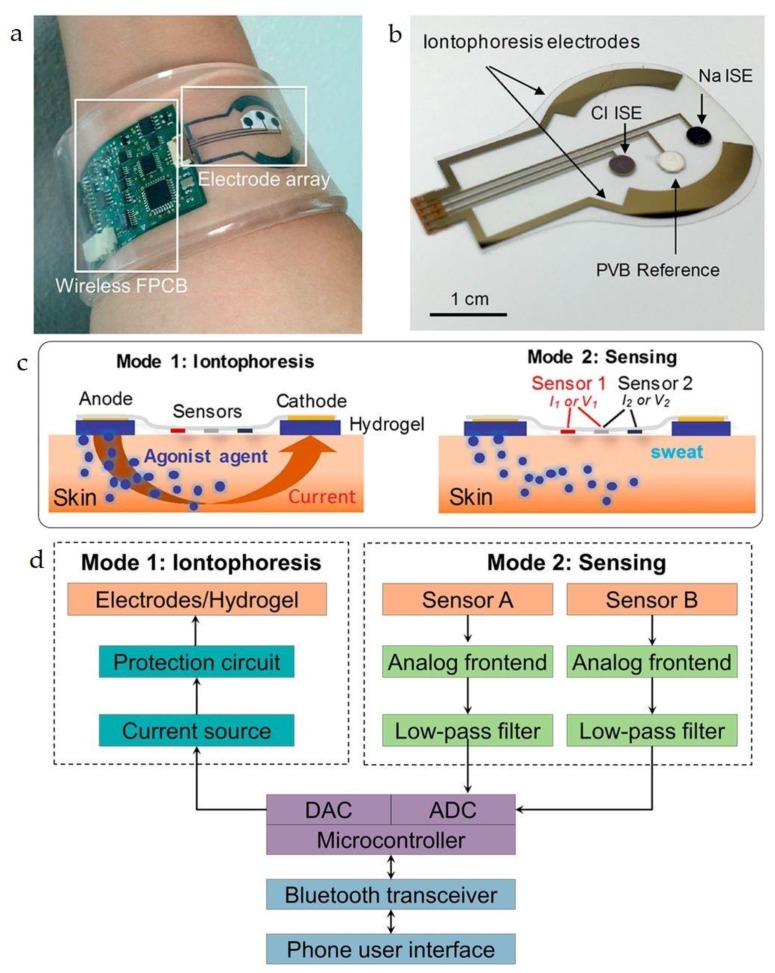
(**a**) Image of the sweat extraction and sensing platform; (**b**) Image of iontophoresis and sweat sensor electrodes for sodium and chloride ion sensing; (**c**) Schematic illustrations of the iontophoresis and sensing modes; (**d**) Block diagram showing the iontophoresis and sensing circuits. Reprinted from [35].

Davis and coworkers demonstrated a method for sweat sampling and analysis, by an iontophoresis interface (see Figure 5), integrated in a wearable sweat analysis platform [35]. 

## 3. Creatinine

### 3.1. Blood Creatinine

Creatinine is produced by the body through muscle activity and is filtered by the kidneys. Therefore, creatinine levels in blood reflect the state of renal function. The normal physiological concentration is in the range 40–150 μM. Values > 500 μM indicate severe renal impairment, while levels <40 μM indicate decreased muscle mass [36]. 

The application of potentiometric sensing for quantitative estimation of creatinine has been widely reported showing good accuracy and miniaturization potential [37]. Many sensors are based predominantly on the hydrolysis of creatinine by creatinine iminohydrolase (CIH). In the presence of creatinine, the CIH enzyme generates ammonia that can be detected by any pH, ammonia gas, or NH_4_^+^ ion-selective electrode. The device is not influenced by the presence of creatine, but suffers interferences from endogenous ammonia. 

Other electrochemical techniques have been also developed for enzymatic detection of blood creatinine. Disposable amperometric biosensors have been reported by Madaras and Buck [38]. The base electrodes were fabricated as a multilayer film on a polyimide foil using micro-fabrication techniques. The multienzyme system (creatininase, creatinase, sarcosine oxidase) was immobilized on top of permselective layer via cross-linking of the proteins with glutaraldehyde. These planar sensors for creatinine and creatine measurement had a fast response time of less than a minute, a linear response up to 1.2 mM in batch-type and 2.0 mM in flow injection analysis, and a lower limit of detection of 10–20 mM. These features are particularly promising for incorporation in portable analyzers.

Wei et al. reported an accurate polymer-based electrochemical POC assay for creatinine level detection from whole blood [39]. The lower limit of detection was ca. 0.46 mg/dL of creatinine with only 40 μL sample and a creatinine concentration range from 0 mg/dL to 11.33 mg/dL. 

### 3.2. Urine Creatinine 

The concentration of creatinine in urine is not influenced by the protein intake. Therefore, unlike urea, it is a more reliable biomarker of renal function. The normal levels for creatinine concentration in urine are in the range from 500 to 1500 μg/mL, respectively, increasing in patients with chronic nephritis or renal dysfunction [40]. 

Main challenges are the correction of matrix interferences because the level of NH_4_^+^ in urine is about the same as the creatinine level (10^−2^ to 10^−3^ M), and also the ionic composition of urine shows larger variability than for blood serum. CIH-based creatinine biosensors, coupled to NH_4_^+^ ion selective electrodes, have reported to have good characteristics for creatinine quantification in urine [41]. 

An enzyme-free preanodized screen-printed electrode (SPE) has been used for the selective and quantitative recognition of creatinine in human urine [42]. Creatinine levels from 0.37 to 3.6 mM were selectively detected by square-wave voltammetry with a slope and regression coefficient of 16.7 μA/mM and 0.998, respectively, and a lower limit of detection (signal/noise = 3) of 8.6 μM. 

**Figure 6:**
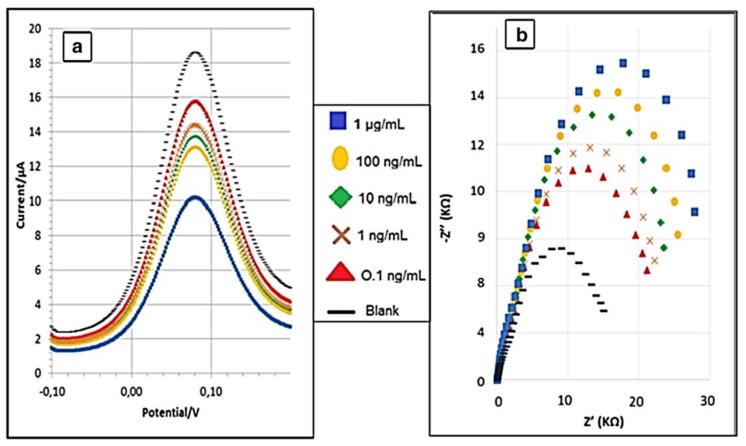
Differential pulse voltammograms of different concentrations of creatinine solutions (**a**) and Nyquist plots of different concentrations of creatinine solutions (**b**) in 5.0 mM[Fe (CN)6]^3−^ and 5.0 mM [Fe (CN) 6]^4−^ in PBS buffer at pH 7.4. Z’ and Z’’ in Figure 6b represent the real and the imaginary part, respectively, of the impedance. Reprinted from [43].

A new creatinine sensor based on a novel molecularly imprinted polymer using screen-printed gold electrodes (Au-SPE), was developed [43]. The reported results indicate that the MIP had a specific recognition ability for creatinine, while other structurally related compounds, such as urea or glucose, could not be recognized on the MIP. The EIS and DPV biosensor responses reported in Figure 6 show a limit of detection of 0.016 ng/mL and 0.081 ng/mL, respectively, with a linear range from 0.1 ng/mL to 1 μg/mL.

In addition, these biosensors were tested on volunteers with different creatinine urine levels validating them as a promising tool for measurement of creatinine in point-of-care devices. This study indicated a promising strategy for the fabrication of sensing devices based on MIP with highly selective recognition ability, simplicity of operation, small size and low cost.

A biomimetic Fe_3_O_4_@PANI NPs sensor based on magnetic field-induced self-assembly was fabricated for the sensitive detection of creatinine. The hydrophilic Fe_3_O_4_@PANI can provide abundant sites, amino groups in PANI, for adsorption of creatinine via formation of hydrogen bonds, and also provide a pathway for electron transfer. Good stability and reproducibility in the determination of creatinine were demonstrated, with a detection limit reaching of 0.35 nmol L^−1^ (S/N = 3) and successfully clinical determination of creatinine in human urine samples with average recoveries between 90.8% and 104.9% and RSD lower than 2.7% [44]. A non-enzymatic electrochemical technique for creatinine sensing was presented by exploiting the iron binding property of creatinine. Disposable carbon printed electrodes layered with FeCl_3_ coated cotton fiber membranes were used to sense creatinine from 10 to 245 mg/dL, on clinical urine samples [45]. 

**Figure 7:**
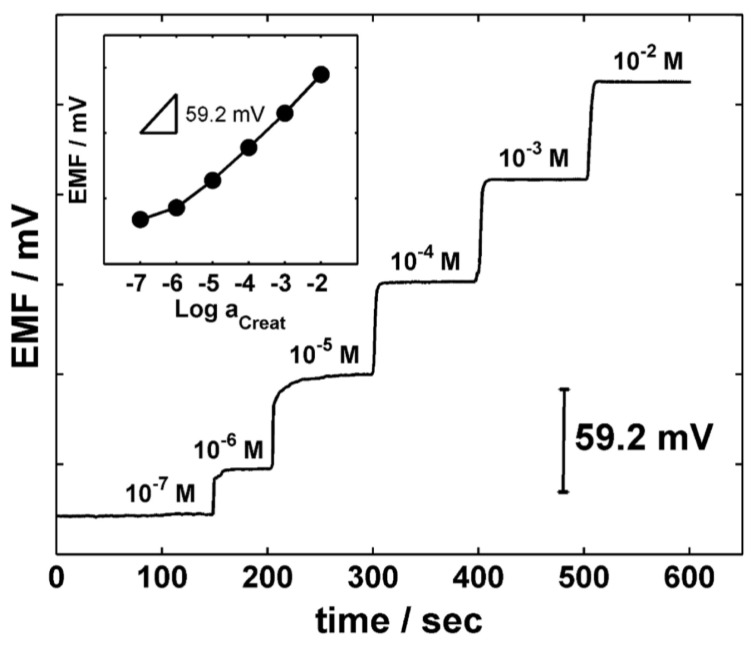
Potential-time plot for different creatinine concentrations of the calix [4] pyrrole-based sensor. Inset is reported the calibration curve (RSD 0.6% for N = 5). Reprinted from [46].

The Andrade group proposed a novel synthesized calix[4]pyrrole-based molecule as an ionophore for the determination of creatininium cations [46]. The sensor is highly selective and very sensitive (Figure 7), allowing to detect creatinine at concentrations much lower than the normal levels in urine or blood. 

Interestingly, the strong response of these sensors to creatininium cations compared to other cations such as K^+^ and Na^+^, which are typically present in higher quantity than the creatininium cation, decrease their interference [47]. 

### 3.3. Sweat Creatinine 

Previous literature reports suggest that the amount of creatinine in sweat fluid is too low to consider sweat as an important excretion route for this biomarker. As a result, the concentration of creatinine in sweat may represent only leakage from the blood and not have sufficient physiological importance [48]. 

## 4. Ammonia 

### 4.1. Ammonia Breath Analysis 

Among POC testing approaches, breath analysis is rapidly expanding [49]. Recent promising results have been obtained using exhaled breath for detection of lung cancer, asthma, respiratory infections, gastro-intestinal diseases and diabetes. In recent years, a number of clinical breath tests have been approved, including airway reactivity/asthma based on NO measurements [50]. 

The measurement of volatile species in the exhaled human breath has the potential to contribute to improve the quality of life of CKD patients by early-stage diagnosis of kidney failure and monitoring of hemodialysis treatment progress and frequency [51]. Since Simenhoff et al. found that ammonia and other volatile amines (trimethylamine and dimethylamine) most probably underlie the fishy odor in uremia [52], this has motivated numerous investigation of the breath profile of patients at different stage of CKD [53]. The studies revealed that the composition of volatile organic compounds (VOCs) in the breath is already affected as renal function is only mildly impaired [54]. Exhaled volatile substances mirror clinical conditions in pediatric chronic kidney disease [55]. However, progress in the use of breath analysis for the diagnostics and treatment monitor in CDK has been slow. Main challenges concerns two main factors, namely (i) the very low concentrations of biomarkers in the exhaled breath, which necessitates of ultrasensitive analyzers; and (ii) the use of complicated sample preparation, time-consuming test procedures, and large size instrumentation such as gas chromatography (GC) and mass spectrometry (MS). To overcome these challenges, chemoresistive sensors [56] and portable hybrid GC/chemical sensor analyzers [57] have been proposed. 

Recent studies are in fact aimed to develop new miniaturized, reliable sensing devices that can detect the desired biomarkers in the ppb-ppt range and overcome other critical issues such as humidity interference and cross sensitivity, which limit the use of breath analysis for medical diagnostics and point-of-care monitoring [58]. In particular, metal oxide semiconductor (MOS) sensors based on tailored nanomaterials such as nanostructured metal oxides, carbon nanotubes (CNTs) or graphene, hybrid organic/inorganic composites have shown the promise to achieve extremely high sensitivity (ppb or lower) requested for breath analysis. This is attributed to their extremely large surface-to-volume ratio, and the possibility to customize their surface via recent nanofabrication approaches [59,60]. 

Amongst the numerous breath components, ammonia has been proposed as the most promising potential biomarker for non-invasive POC testing. In CKD patients, the accumulated urea is degraded by salivary urease into ammonia, which is then excreted by breathing. Breath ammonia can therefore be used for detecting the increased nitrogen-containing wastes. In a pilot study, an electrochemical sensor was used to measure breath ammonia in healthy volunteers and patients with CKD [61]. Breath ammonia can be used to recognize oxidative stress, metabolic conditions and haemolysis during HD [62]. 

Measurement of ammonia levels in real breath samples require, however, highly sensitive gas sensors capable to measure concentrations from 50 ppb to several ppm. Further, as the levels of breath NH_3_ are significantly influenced by the patient’s oral condition including urease activity and salivary pH, great care should be taken in collecting and analyzing the sensor data [63]. 

**Figure 8:**
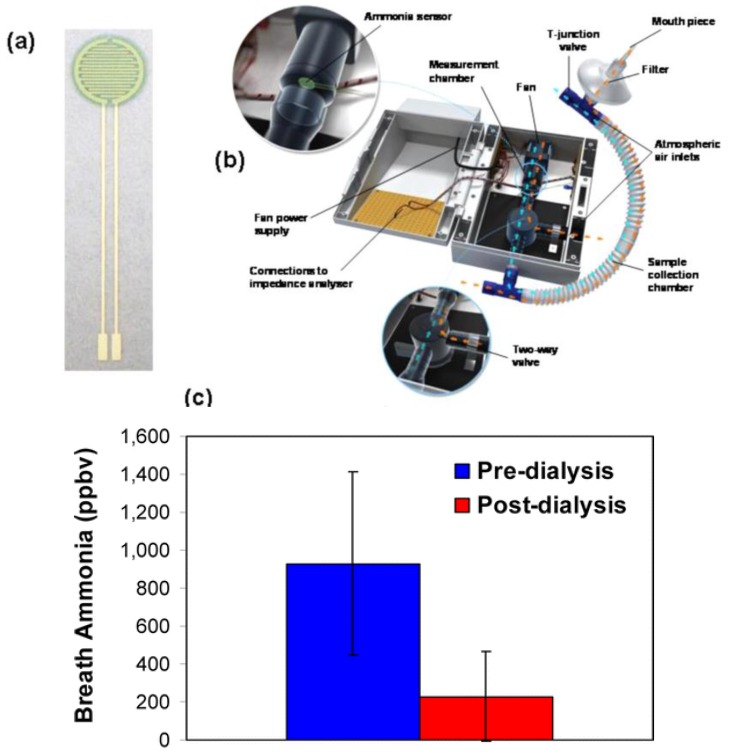
(**a**) Inkjet-printed ammonia sensor; (**b**) POC device for measuring breath ammonia levels; (**c**) Breath ammonia measured in patients with end-stage kidney disease before and after dialysis. Reprinted from [65].

Limeres et al. validated a breath ammonia measurement method for monitoring patients with end-stage renal disease, showing a strong correlation between breath ammonia and blood and saliva ammonia during both pre- and post-dialysis [64]. A POC device for measuring human breath ammonia was also developed by Hibbard et al. based on a disposable NH_3_ sensor (Figure 8a,b) containing PANI nanoparticles [65]. Notably, these sensors can measure breath ammonia from 40 to 2993 ppbv (*r*^2^ = 0.99, *n* = 3). Measurement of ammonia in the breath of patients with end-stage kidney disease revealed its significant reduction following dialysis (Figure 8c). 

Excellent intra-individual correlations were also demonstrated between breath ammonia and BUN, confirming previous results [66]. The results demonstrated the possibility of using POC breath ammonia systems as a noninvasive means of monitoring kidney dysfunction and treatment progress. 

**Figure 9:**
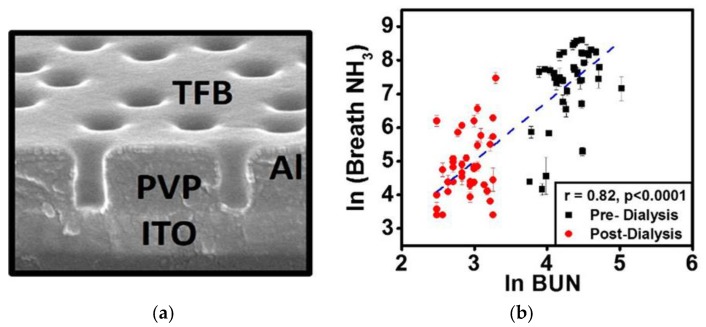
(**a**) Cylindrical nanopore structure of poly[(9,9-dioctylfluorenyl-2,7-diyl)-co-(4,4′-(*N*-(4-s-butylphenyl))diphenylamine)] (TFB) sensing material; (**b**) Analysis of the breath profile of patients before and after dialysis. Reprinted from [67].

Chuang et al. reported an organic-based sensor system to monitor breath ammonia during hemodialysis. The system was based on a poly[(9,9-dioctylfluorenyl-2,7-diyl)-co-(4,4′-(*N*-(4-s-butylphenyl))diphenylamine)] (TFB) sensor with a cylindrical nanopore structure exhibiting a high sensitivity to ammonia in the ppb-regime (Figure 9). 

Analysis of the breath profile of patients before and after dialysis revealed a good correlation with blood ammonia and a clear drop after dialysis suggesting the potential use of these low-cost device for daily tracking of hemodialysis patients [67]. Ishida et al. demonstrated the continuous measurement of breath ammonia concentration in patients undergoing dialysis treatment using a quartz crystal microbalance (QCM) sensor. It was shown that ammonia decreased gradually as the treatment proceeded with a strong correlation between BUN and NH3 in expired gas. Furthermore, strong correlation were observed between changes in the frequency of the QCM gas sensor and both the pre-dialysis BUN level (*r* = 0.71, *p* < 0.05) and the post-dialysis BUN level (*r* = 0.90, *p* < 0.05) indicating that miniaturized QCM systems may be utilized to track progress of dialysis treatment [68]. 

### 4.2. Blood Ammonia

**Figure 10:**
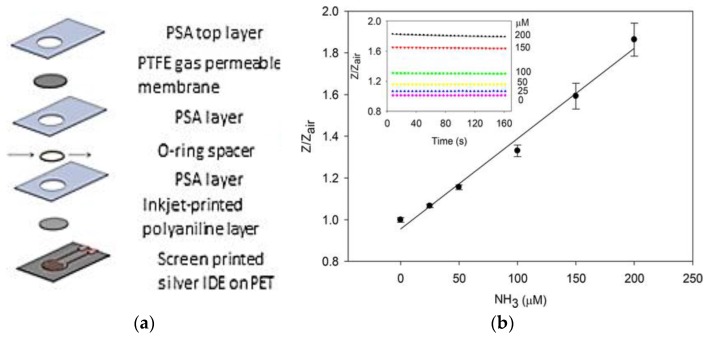
(**a**) Description of devices; (**b**) Calibration curve. Reprinted from [69].

Blood ammonia levels are relevant to a number of medical conditions. Physiological levels of ammonia in blood are in the range 11 to 50 µM. Higher ammonia levels in blood represent a serious medical emergency and can lead to significant and permanent neurological impairment if not addressed quickly. A POC device (Figure 10) for sensitive, low volume measurement applications of blood ammonia has been proposed by Killard’s group [69]. 

An electrochemical sensors for measuring blood ammonia was also developed by the same group [14,70]. The device require a low serum sample and measure impedance change with respect to air at 1 kHz, 5 mV rms. The device was capable of the measurement of ammonia in serum across the physiologically relevant range of 25–200 µM (*r*^2^ = 0.9984) and had a limit of detection of 12 µM (*n* = 3). The device showed no significant cross-sensitivity to common electrochemical interferences components present in blood. Devices displayed minimal variation over time (0.64%) with respect to their impedance in air (*n* = 12) and could be stored in desiccant for at least five months.

### 4.3. Urine Ammonia

Traditional practical and technical limitations of urine ammonia measurement have impeded the use of urine ammonia as a routinely employed biomarker. However, projects focused on the development of analyzer technologies directed at ammonia measurement and their implementation into a practical handheld device prototype are currently explored. Liu et coworkers, proposed a technology based on a unique hybrid sensor array capable of quick, precise, and reliable measurement for ammonia quantification in urine samples in real-time scale. The miniaturized, low cost, easy to use handheld device prototype will utilize solid-state electronics with a high level of integration. The analyzer will be capable of being applied in two broad formats: (1) a handheld device for outpatient or inpatient applications requiring less frequent (e.g., daily) monitoring; and (2) a urinary catheter device for semi-continuous measurement for inpatient applications requiring intensive serial monitoring [71]. 

### 4.4. Sweat Ammonia

Plasma ammonia is the principal source of ammonia in sweat [72]. Ammonia and ammonium in blood are mostly a result of metabolic degradation of proteins. The difference between the pH value of sweat (4.0–6.8) and blood (7.35–7.45) induce a pH gradient. Ammonia diffuses from a higher to a lower pH value, i.e., from blood to sweat. The size of ammonia molecules is similar to water molecules. As such, they are permeable through cell membrane.

Then, sweat ammonia sensor offers considerable promise for monitoring metabolic disorders in healthcare. Guinovart et al. presented a novel ion-selective potentiometric cell in a temporary-transfer tattoo platform for monitoring ammonium levels in sweat [73]. The fabrication of this skin-worn sensor, based on a screen-printed design, incorporates all-solid-state potentiometric sensor technology for both the working and reference electrodes, in connection to an ammonium-selective polymeric membrane based on the non-actin ionophore. The resulting tattooed potentiometric sensor exhibits a working range between 0.1 mM and 0.1 M, which is well within the physiological levels of ammonium in sweat. Testing under stringent mechanical stress expected on the epidermis shows that the analytical performance is not affected by factors such as stretching or bending. Such a combination of the epidermal integration, screen-printed technology and potentiometric sensing represents an attractive path towards non-invasive monitoring of a variety of electrolytes in human perspiration.

## 5. Challenges and Perspectives

The market for POC testing is expanding as the number of people suffering from chronic diseases is increasing rapidly, due to the aging of our populations. Developers of POC testing devices face strict design criteria and a growing number of potential applications. First, POC testing should provide all the functions of centralized laboratory testing while eliminating the need for trained staff. POC testing offers now more flexibility to meet a diverse range of medical needs because small, portable POC devices make testing possible in a variety of remote locations, such as underserved populations, rural areas, and locations with limited infrastructure or personnel. However, there are still many challenges associated with POC devices, and especially regarding their practical implementation. POC testing should provide reliable quantitative results, their easily comprehensible presentation, simple decision support and, ideally, connectivity to other information systems such as the patient’s electronic health record. Another key requirement is that POC testing give consistent results with established laboratory analysis methods. 

As discussed above, several new biosensors have been developed, which can be miniaturized and are highly attractive for the POC market for CDK patients as they do not require reagents and can be used with different, often easily accessible, body fluids. 

Portable and handheld POC devices to measure renal disease markers are already present in the marketplace. For instance, StatSensor (Nova Biomedical, Waltham, MA, USA) and i-STAT (crea-cartridge, Abbott Point of Care Inc., Princeton, NJ, USA), utilize enzymatic reactions to detect creatinine electrochemically. While, the i-Stat device has a 100 μL volume requirement, which makes it challenging for finger-prick sampling, the StatSensor is a simple device with considerable potential that can measure creatinine from a finger-prick sample. Despite these promising achievements, the development of POC sensing technologies remains challenging and significant further research efforts are required. Notably, interference with several of the components found in body fluids, such as glucose, fructose, ketone bodies, ascorbic acid, may affect the accuracy of the measurement. Using antibody-based sensors rather than enzymatic ones, interfering reactions and signals can be minimized even when working with a complex matrix such as whole blood. 

**Figure 11:**
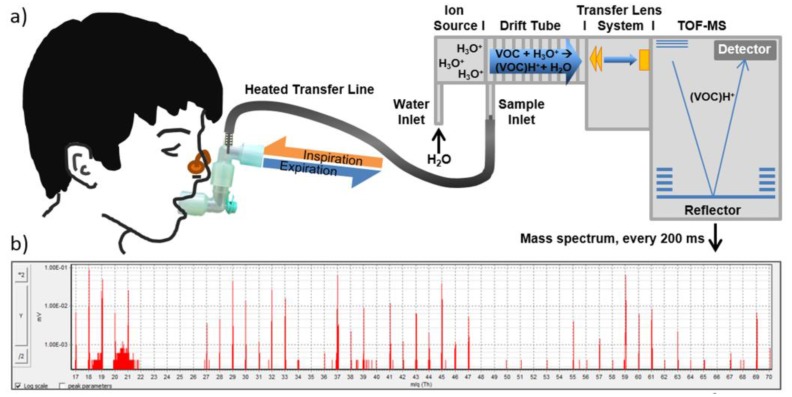
Schematic description of continuous real-time breath analysis system. (**a**) Participants breathed through a sterile mouthpiece without resistance. Ex- and inhaled breath was transferred continuously into the heated transfer line (connected via t-piece) of the PTR-ToF-MS in a side-stream mode at a flow of 20 mL/min; (**b**) Every 200 ms a TOF—mass spectrum was recorded. Reprinted from [55].

Moving away from the analysis of blood or urine, it is possible that future POC may involve gas molecule sensing which detects changes in the volatile compounds produced in the breath [74]. A recent investigation of the exhaled volatile substances (see Figure 11) in pediatric subjects suffering of mild-to-moderate CDK revealed that ammonia, ethanol, isoprene, pentanal and heptanal had about twice as high levels than in healthy subjects, while methylamine was lower [55]. Furthermore, it was observed that while ammonia accumulated already in CKD stage 1, alteration in the concentration of pentanal, heptanal and isoprene were detectable in stage 2 to 4 possibly providing a non-invasive approach to monitor disease progress. 

A focused analysis of the VOC breath and blood profile of end-stage renal disease patients revealed that six compounds, namely isoprene, dimethyl sulfide, methyl propyl sulfide, allyl methyl sulfide, thiophene and benzene, changed their blood and breath level during hemodialysis treatment [53]. It was shown that volatile sulphur compounds were removed during hemodialysis suggesting their potential use in conjunction to breath ammonia measurement for monitoring of sufficient dialysis dose. A major challenge remains the interpretation of the VOCs patterns as numerous external factors, not directly associated with the disease, play an important role in their composition. For instance, contaminants from extracorporeal circuits and hospital room air during hemodialysis have shown to significantly influence the concentration of numerous volatile species in both the blood and breath patterns of CDK patients. 

Demirjian compared the exhaled breath of subjects with end stage renal disease to healthy volunteers to identify volatile compounds that can serve as a potential breath-print for renal failure [54]. Using a random forests classification model, they identified three volatile species, namely 2-propanol, ammonia and acetaldehyde that were highly significant for discriminating individuals with renal failure from individuals without renal failure (C statistic > 0.99). Lipophilic protein-bound toxins are increasingly investigated as potentially responsible for several biochemical and functional alterations in uremia [75]. As the alveolar capillary membrane is highly permeable for lipophilic substances, the breath profile is expected to contain reliable information on their blood levels and may serve a facile approach for their clearance monitoring. Further research is focusing on adding additional sensors for measurement of further ions besides sodium and chloride and even metabolites like lactate, creatinine, urea and glucose. This data could be used to predict many health conditions and diseases like muscle injury, kidney disease or diabetes [76]. 

Besides new biomarkers discover is performed by using lab instrumentation, in the future we foresee that small specific sensors for volatiles of interest will be incorporated into small portable or handheld devices for POC. Electronic nose technology is now being refined and adapted for diagnostic applications [77]. Achieving selective sensing of ppb and ppt level of CKD biomarkers remains a key research direction, due to the complex composition of human breath.

Further, miniaturization of the sensing system is another key research priority. Advanced techniques for manufacturing small and portable sensors for point of care diagnostics are actively searched [78]. Inkjet printing is a promising alternative manufacturing method to conventional standard microfabrication techniques for the development of flexible and low-cost devices. The use of flexible plastic substrates and biocompatible inks, as well as the rapid prototyping and low-cost of the fabricated sensors, open new opportunities in the field of POC device design. 

All these emerging scientific and technological innovations, coupled with lab-on-a-chip, wearable devices and enhanced connectivity, are expected to transform the point of care diagnostic perspectives in the next future [79]. POC tests performed with these miniaturized portable devices, when implemented properly and thoughtfully, can have a positive impact on operational efficiency and patient care. They can become key parts of a point of care health model, which through an embedded local unit (gateway) for sensor data acquisition-processing-communication and a remote e-Health service center, can be integrated and scaled in different telemedicine scenarios [80].
